# Rapid deterioration of Mooren’s ulcers after conjunctival flap: a review of 2 cases

**DOI:** 10.1186/s12886-017-0488-1

**Published:** 2017-06-15

**Authors:** Saiqun Li, Yuqing Deng, Caiyuan Du, Haixiang Huang, Jing Zhong, Ling Chen, Bowen Wang, Jin Yuan

**Affiliations:** 1 State Key Laboratory of Ophthalmology, Zhongshan Ophthalmic Center, Sun Yat-sen University, Guangzhou, 510060 China; 2 Allad Eye Hospital, Zhanjiang, 524043 China

**Keywords:** Mooren’s ulcer, Conjunctival flap, Keratoplasty, Case report

## Abstract

**Background:**

Conjunctival flaps are a widely used treatment for numerous corneal ulcers that are caused by microorganismal infections. However, whether it can be performed on immune-mediated corneal ulcers is controversial.

**Case presentation:**

We present two cases of Mooren’s ulcer that were treated using conjunctival flap in an attempt to prevent further corneal perforation at their local hospital. A rapid acceleration in ulcer progression was observed after a conjunctival flap was applied. Ultimately, the two patients underwent corneal transplantation, which required the postoperative use of topical immunosuppressants and resulted in a final cure. In the current report, we also discussed this incorrect surgical choice via a review of conventional interventions that are used to treat Mooren’s ulcer.

**Conclusions:**

These two cases demonstrate that keratoplasty combined with topical immunosuppressants is effective in treating Mooren’s ulcer. Application of conjunctival flaps or autografting could promote progression of ulceration in Mooren’s ulcers.

## Background

Mooren’s ulcer is a relentless, painful, and chronic ulcerative keratitis that was first described by Bowman in 1849 and later by Mooren in 1867 [1–3]. It is a rare and potentially blinding ocular disease that is more commonly observed in the African, China and India [4]. Although its underlying etiology remains unclear, it is widely accepted that Mooren’s ulcer is an idiopathic autoimmune disease [4–6]. A typical Mooren’s ulcerative lesion is characterized by an overhanging edge and stromal melting. The ulceration begins in the peripheral cornea and tends to progress circumferentially or centrally, whereas rarely progressing to involve the entire cornea [6, 7]. The natural progression of Mooren’s ulcer varies substantially, and corneal melting may cause descemetoceles or, in severe cases, corneal perforations [8]. Conventional interventions for Mooren’s ulcer include the local or systematic use of steroids or non-steroidal immunosuppressants, conjunctival resection, lamellar keratoplasty (LKP), penetrating keratoplasty (PKP), epikeratoplasty or amniotic membrane transplantation [3, 4, 8–11]. Conjunctival flap has been confirmed to be a simple, well-supported, short-term treatment for managing corneal perforation or impending perforation in infective corneal ulcers [12–14]. However, it is not recommended for the Mooren’s ulcer, a peripheral autoimmune-related ulcerative corneal diseases. In the current report, we present two patients with Mooren’s ulcer who were incorrectly subjected to conjunctival flap to prevent corneal perforation. In each of these two cases, a rapid deterioration was observed in the ulceration following conjunctival flap.

## Case presentation

### Case 1

**Fig. 1:**
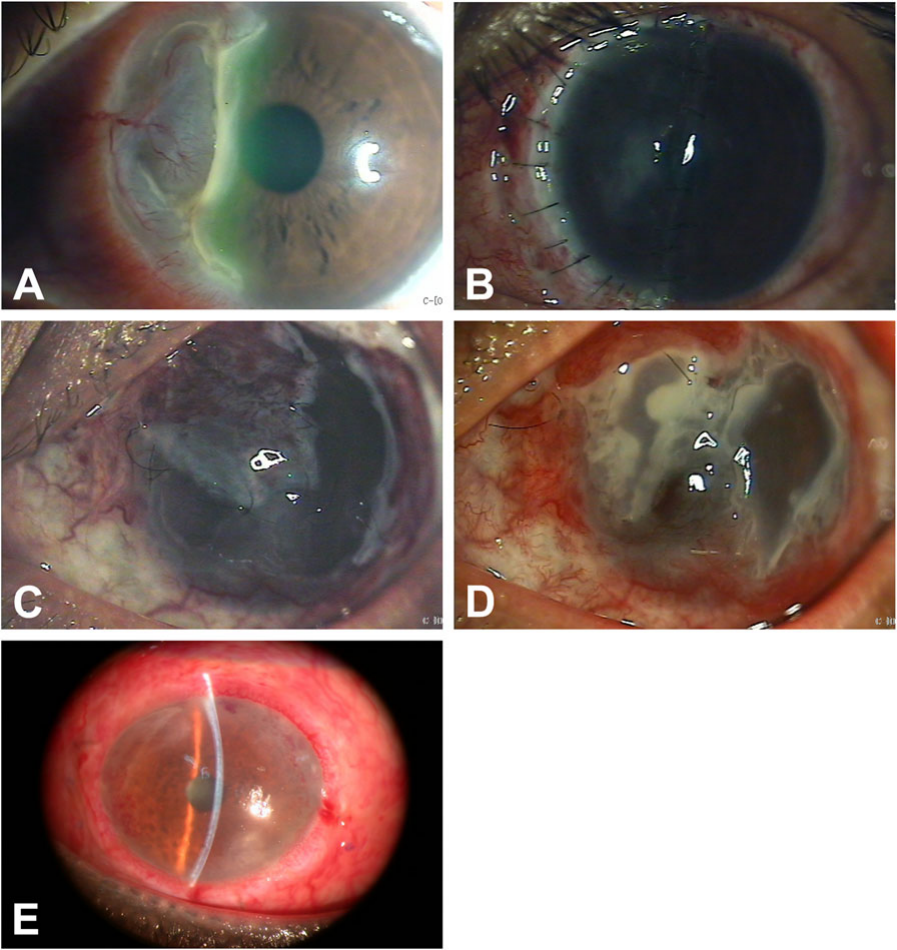
The progression of Mooren’s ulcer in case 1. **a** A slit-lamp examination revealed a peripheral corneal ulcer that ranged from 6:00 o’clock to 11:00 o’clock and presented a grey, steep, overhanging leading edge. **b** The patient underwent “D” shaped lamellar keratoplasty. **c**, **d** Ulceration rapidly progressed circumferentially and centrally after conjunctival flap was performed. **e** The patient eventually underwent a full lamellar keratoplasty

**Fig. 2:**
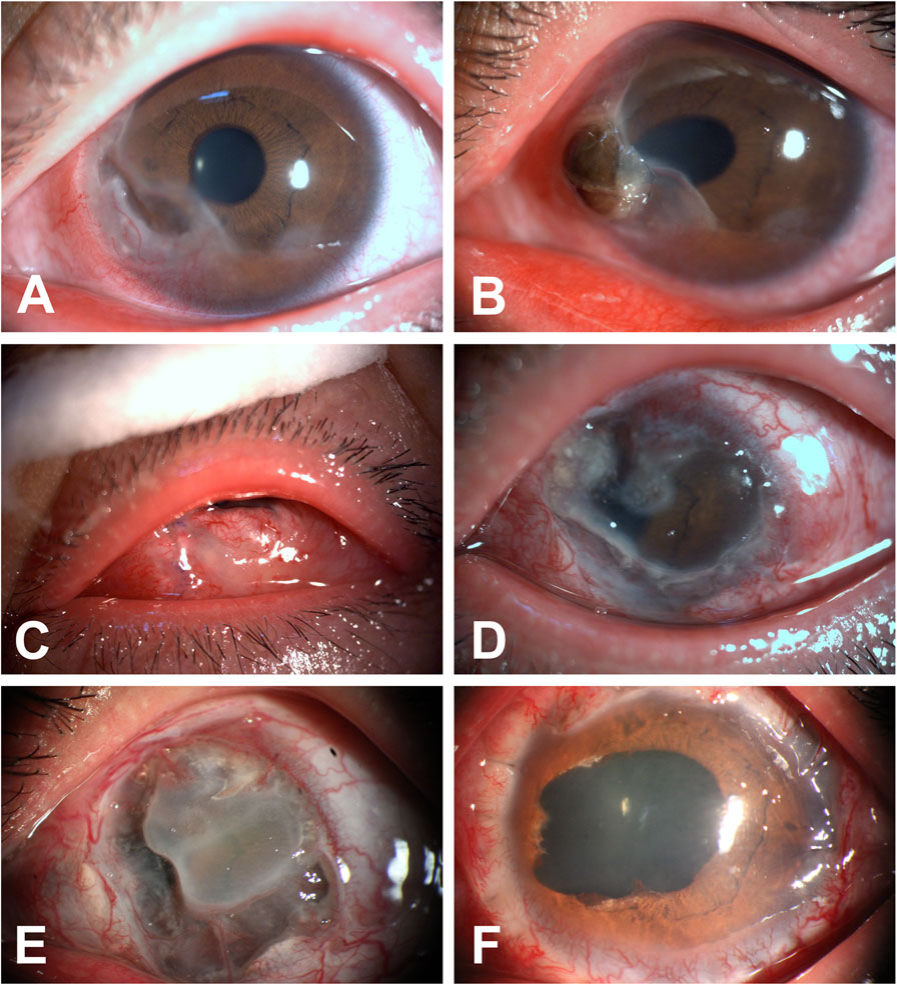
The progression of Mooren’s ulcer in case 2. **a** A slit-lamp examination revealed a peripheral ulcer with a typical steep and overhanging leading edge. **b** Corneal perforation subsequently occurred in the ulcerative lesion. **c** The patient underwent conjunctival flap to prevent the progress of the corneal lesion. **d**, **e** Ulceration rapidly progressed circumferentially and centrally after conjunctival flap was performed. **f** The patient eventually underwent a full penetrating keratoplasty

A 29-year-old man complained of pain and progressive blurred vision in his left eye for one month. Initially, the patient visited the local hospital and was administered antibiotic eye drops (tobramycin 0.3%, Alcon-Couvreur, Belgium), nonsteroidal anti-inflammatory eye drops (diclofenac sodium 1%, Xingqi Pharma, China) and steroid eye drops (fluorometholone 0.1%, Santen Pharma, China) without obvious relief. He denied any history of ocular trauma, ocular surgery, diabetes, parasitic infections, rheumatoid arthritis and other associated diseases. His uncorrected visual acuity was 20/20 in the right eye and 20/40 in the left eye. A slit-lamp examination of his left eye revealed a peripheral corneal ulcer from 6:00 o’clock to 11:00 o’clock that had a grey, steep, overhanging leading edge that reached the central optical zone of the cornea (Fig. 1a). The conjunctiva adjacent to the corneal ulcer was severely congested, with new superficial big to small-sized blood vessels extending into the corneal ulcer area from the limbus. Within the ulcer, the thinnest area was less than 20% of the normal corneal thickness. An examination of the anterior segment of his right eye showed that it was normal. A systematic examination and laboratory tests were performed to rule out infectious keratitis, rheumatoid arthritis and other immune-associated diseases. Based on the clinical characteristics and medical history of the patients, a Mooren’s ulcer was diagnosed. To prevent further corneal perforation, lamellar keratoplasty (LKP) was conducted in his local hospital. A “D” shaped lamellar graft was designed to correspond to the shape of the ulcer (Fig. 1b). A topical antibiotic and steroid (tobramycin 0.3%/dexamethasone 0.1%, Alcon-Couvreur, Belgium) were administrated postoperatively. One month later, the Mooren’s ulcer had slightly recurred at the graft, and following treatment, a conjunctival flap was introduced in the patient by his local ophthalmologist. About several days after the performance of conjunctival flap, the ulceration rapidly progressed circumferentially and centrally, with a small island containing a transparent area eventually left in the temporal cornea (Fig. 1c, d). The patient ultimately underwent a full LKP in our hospital (Zhongshan Ophthalmic Center, ZOC) (Fig. 2e). Postoperatively, tobramycin 0.3%/dexamethasone 0.1% eye drops and ointment (Alcon-Couvreur, Belgium), Tacrolimus 0.05% eye drops (prepared by ZOC) and sodium hyaluronate 0.3% eye drops (Alcon-Couvreur, Belgium) were administered. There was no recurrence for one year following the second keratoplasty.

### Case 2

A 21-year-old female presented to the local hospital with 5 days of pain, photophobia and tearing in her left eye. A slit-lamp examination using fluorescence staining revealed a grey-white infiltration in the periphery of the cornea from 4:00 o’clock to 7:00 o’clock. The conjunctiva and episclera adjacent to the involved area were severely congested, and a fascicle of new blood vessels extended to the corneal infiltration. Her right eye was normal. She was diagnosed with viral keratitis and treated with levofloxacin 0.5% eye drops (Santen Pharma, China) and α2b interferon eye drops (Anhui Anke Biotech, China). One month later, she reported no obvious relief and her local ophthalmologist corrected the original diagnosis to fascicular keratitis. She was then administrated tobramycin 0.3%/dexamethasone 0.1% eye drops and ointment (Alcon-Couvreur, Belgium) and sodium hyaluronate 0.3% (Alcon-Couvreur, Belgium), and she subsequently reported occasional relief. At eight months from the onset of her condition, the corneal lesion slowly progressed and she eventually developed a large corneal ulcer with a steep and overhanging leading edge (Fig. 2a). After diabetes, parasitic infections, rheumatoid arthritis and other associated systematic diseases were ruled out, a diagnosis of Mooren’s ulcer was made. The cornea within the ulcerated area continued to slowly thin, and a perforation occurred during her hospital stay (Fig. 2b). To avoid a progressive corneal lesion, conjunctival flap was performed in her local hospital (Fig. 2c). However, the ulceration rapidly expanded to encompass the entire cornea, and severe vessel engorgement was postoperatively observed in the conjunctival graft (Fig. 2d, e). The patient eventually received a full penetrating keratoplasty (PKP) in our hospital (Zhongshan Ophthalmic Center, ZOC) (Fig. 2f) and was postoperatively administered tobramycin 0.3%/dexamethasone 0.1% eye drops and ointment (Alcon-Couvreur, Belgium), Tacrolimus 0.1% (Senju Pharma, Japan) and sodium hyaluronate 0.3% (Alcon-Couvreur, Belgium). There was no recurrence for four months after keratoplasty.

## Discussion and conclusions

Mooren’s ulcer is an idiopathic, progressive, and painful peripheral ulcerative keratitis that occurs in the absence of any other associated systematic diseases [6]. To our knowledge, the etiology of Mooren’s ulcer remains unclear, but there is a general consensus that it may have an immunological basis [4–6, 15–17].

Most experts advocate “the stepladder approach” for managing Mooren’s ulcer [4, 6, 18]. Representative studies in the literature that describe interventions for Mooren’s ulcer are listed in Table 1 [3, 8, 11, 19–27]. Conventional therapies involve the topical or Systematic application of steroids (such as dexamethasone and prednisolone) and immunosuppressive reagents (such as cyclosporin A and tacrolimus (FK506)) [8, 19, 28, 29], and surgical treatments (such as conjunctival resection, freezing or thermocoagulating) [4, 6, 11]. Occasionally, Mooren’s ulcers respond poorly to the conventional therapies that have been described here, and these patients may be at risk of corneal perforation [8]. In these cases, LKP or PKP should be introduced [8, 23, 24]. However, currently, the lack of donated corneas remains a major issue all over the world, especially in some Asian and African counties [30–32]. In case of numerous other corneal diseases, including corneal perforation, infectious keratitis and resistant corneal ulcers, the conjunctival flap can been used as an alternate procedure. Conjunctival flaps are helpful for maintaining ocular integrity, reducing corneal pain, reducing inflammatory processes, arresting corneal ulceration and preventing secondary infection [12, 33]. However, can the conjunctival flap procedure be applied in patients with Mooren’s ulcers?

**Table 1:** Representative studies describing interventions for Mooren’s ulcers

References	Type	Interventions	Effective
Lal (2015) [11]	Case series	Conjunctival resection + systemic immunosuppression	√
Chen et al. (2000) [8]	Case series	Topic cyclosporin A + LKP	√
Liu et al. (2015) [23]	Case series	Modified LKP + immunosuppressive therapy	√
Xie et al. (2006) [24]	Case series	Topic tacrolimus alone or + KP	√
Ngan et al. (2011) [25]	Case series	AMT	√
Schallenberg et al. (2013) [27]	Case series	AMT alone or + conjunctival excision	×
Alhassan et al. (2014) [4]	Systematic review	Conjunctival flap	N/A
Brightbill et al. (2008) [21]	Textbook	Conjunctival flap	N/A
Chen et al. (2004) [22]	Case report (One case)	Conjunctival flap + AMT (excised from opposite healthy eye)	Caused replasing
Our observation	Case report (Two cases)	Conjunctival flap (excised from the same diseased eye)	Caused rapid deterioration

*LKP* lamellar keratoplasty, *KP* keratoplasty, *AMT* amniotic membrane transplantation, *N/A* not applicable

A search of databases revealed that a few studies and textbooks mentioned conjunctival flap being used to treat Mooren’s ulcers [4–6, 21, 34]. However, probably because it was performed very rarely and chosen only in some extenuating circumstances, we were not able to retrieve the rate of successful treatment. In our study, we describe two cases of Mooren’s ulcer in which conjunctive flap (excised form the same diseased eye) was performed for management of corneal perforation since donor corneas were not available at the time. However, conjunctive flap appeared to cause a rapid ulceration deterioration and a subsequent expansion to the entire cornea. Our report lends further supportive to the findings of Chen [22]. Chen and associates previously observed a Mooren’s ulcer that relapsed in one patient after amniotic membrane grafting was combined with conjunctival flap (excised from the opposite healthy eye).

There is evidence suggesting that Mooren’s ulcer is an autoimmune disease that is probably directed against a specific antigen in the corneal stroma and triggered by specific risk factors, including a history of ocular surgery or trauma and exposure to viral or parasitic infections [4–6, 15–17]. Both cell-mediated and humoral immune components were shown to be directly involved in the pathogenesis of Mooren’s ulcer [5, 6]. First, immunological aberrations in Mooren’s ulcers appear to involve a subpopulation of T lymphocytes that are known as suppressor T cells (T_S_ cells) [5]. Second, studies have also found circulating IgG antibodies in the cornea and conjunctiva of patients with Mooren’s ulcers [35]. Finally, high levels of serum IgA/IgM and circulating immune complexes have also been detected in patients with Mooren’s ulcers [5, 17].

These alterations in systemic immunity cause a local immune response. The conjunctiva is a blood vessel-enriched tissue. A histopathological study of Mooren’s ulcers revealed that the conjunctival tissues adjacent to the ulcerative cornea housed a large amount of immune effect cells and circulating corneal-associated autoantibodies [6, 26]. The triggering of local autoimmune processes further induced the conjunctiva to produce cornea-destroying enzymes, including collagenase and proteolytic enzymes [15]. Therefore, the conjunctiva serves as a nest for immune mediators and proteases during the pathogenesis of Mooren’s ulcers, which may explain why Mooren’s ulcers always begin in the peripheral cornea. This may also explain why Mooren’s ulcer can be successfully treated using conjunctival resection, freezing and thermocoagulation: because these therapies move activated inflammatory components and proteases away from the cornea, which consequently arrests the progression of the ulceration. However, conjunctival flap, especially when the flap is excised from the same diseased eye, as was performed in the cases described in this study, are in direct opposition to conjunctival resection. Bring the conjunctiva to cover the ulcer will lead to inflammatory mediators and proteases increasing, resulting in ulcer deterioration and progression.

In the two case studies reported here, both patients ultimately underwent keratoplasty and obtained a final cure. Currently, LKP has become the dominant surgical treatment of choice for Mooren’s ulcer [8, 36]. However, in cases such as the second patient in our report in whom corneal perforation has occurred, PKP should be performed instead. Because immunological memory is a hallmark of immune system functions, keratoplasty should be combined with the concurrent administration of topical or systemic immunosuppression to avoid recurrence and graft failure, even when the active disease has been arrested [4, 6]. The reported final healing rate in Mooren’s ulcers that were treated using LKP plus topical immunosuppressive reagents (including 1% cyclosporin A and 0.05%/0.1% Tacrolimus) varied from 89.0% to 100% [8, 29].

In light of our findings, keratoplasty combined with immunosuppression is effective in arresting Mooren’s ulcer and prevent its recurrence. Conversely, conjunctival flap could activate autoimmune process and result the deterioration and progression of ulceration, especially when inflammation has not been controlled. However, it remains unclear whether conjunctival flap could be recommended to treat corneal perforation due to other immune-mediated corneal melting diseases, such as rheumatoid arthritis-associated corneal ulcers and Wegener’s granulomatosis [37].

## Abbreviations

AMT: Amniotic membrane transplantation
LKP: Lamellar keratoplasty
PKP: Penetrating keratoplasty
